# Neural basis of topographical disorientation in the primate posterior cingulate gyrus based on a labeled graph

**DOI:** 10.3934/Neuroscience.2022021

**Published:** 2022-09-09

**Authors:** Yang Yu, Tsuyoshi Setogawa, Jumpei Matsumoto, Hiroshi Nishimaru, Hisao Nishijo

**Affiliations:** 1 System Emotional Science, Faculty of Medicine, University of Toyama, Toyama, Japan; 2 Research Center for Idling Brain Science (RCIBS), University of Toyama, Toyama, Japan

**Keywords:** non-human primate, posterior cingulate gyrus, topographical disorientation, virtual navigation, labeled graph

## Abstract

Patients with lesions in the posterior cingulate gyrus (PCG), including the retrosplenial cortex (RSC) and posterior cingulate cortex (PCC), cannot navigate in familiar environments, nor draw routes on a 2D map of the familiar environments. This suggests that the topographical knowledge of the environments (i.e., cognitive map) to find the right route to a goal is represented in the PCG, and the patients lack such knowledge. However, theoretical backgrounds in neuronal levels for these symptoms in primates are unclear. Recent behavioral studies suggest that human spatial knowledge is constructed based on a labeled graph that consists of topological connections (edges) between places (nodes), where local metric information, such as distances between nodes (edge weights) and angles between edges (node labels), are incorporated. We hypothesize that the population neural activity in the PCG may represent such knowledge based on a labeled graph to encode routes in both 3D environments and 2D maps. Since no previous data are available to test the hypothesis, we recorded PCG neuronal activity from a monkey during performance of virtual navigation and map drawing-like tasks. The results indicated that most PCG neurons responded differentially to spatial parameters of the environments, including the place, head direction, and reward delivery at specific reward areas. The labeled graph-based analyses of the data suggest that the population activity of the PCG neurons represents the distance traveled, locations, movement direction, and navigation routes in the 3D and 2D virtual environments. These results support the hypothesis and provide a neuronal basis for the labeled graph-based representation of a familiar environment, consistent with PCG functions inferred from the human clinicopathological studies.

## 1. Introduction

The retrosplenial complex [the retrosplenial cortex (RSC, areas 29 and 30) and adjacent medial wall of the parietal lobe, including the posterior cingulate cortex (PCC) (areas 23 and 31)] or RSC is implicated in spatial navigation in familiar environments [1],[2]. Human functional activation studies have reported that activity in the posterior cingulate gyrus (PCG), which includes both the RSC and PCC, increases during various navigation tasks [3]–[7], especially during route planning or spontaneous route changes [8],[9], and that its activity is dependent on the stability of landmarks [10],[11]. Furthermore, fMRI studies reported that the PCC is involved in the planning of navigation and coding of distance to goals [12] and suggest that the RSC and PCC form a functional unit (i.e., retrosplenial complex) to code environments [1]. On the other hand, patients with lesions in the PCG, including the RSC and PCC, display topographical disorientation (deficits in wayfinding); they cannot navigate in familiar environments because of their difficulty in orienting themselves relative to known landmark points [13],[14]. In rodents, RSC lesions lead to navigation disturbances during the performance of various spatial paradigms [15]–[17]. These findings suggest that PCG lesions disturb allocentric spatial representations or topological maps to find the right route to a destination [1],[14].

Unit recording studies in rodents support this idea. In rodents, RSC neurons encode various factors that are important for navigation, including head direction, idiothetic information (vestibular cues), and places [18]–[21], as well as landmarks or local environmental cues, specific behaviors during navigation, and running speed [18],[20]–[23]. Furthermore, the activity of RSC neurons reflects complex combinations of local and global positions and turning movements when animals navigated W-shaped tracks [24], route subspaces, and traveled distances on a plus track [25], as well as routes in a T-maze [22],[26]. However, neuronal sensitivity during navigation in the PCC (areas 23 and 31) is unknown in most neurophysiological studies since the rodent brain lacks areas corresponding to primate areas 23 and 31 [2]. In monkeys, it is reported that PCC neurons code target locations on a screen based on allocentric coordinates [27]. The available data suggest that the PCG integrates various factors or items that animals encounter while navigating along a route, which might contribute to planning or selection of routes [8],[22],[26].

It is noted that patients with topographical disorientation cannot navigate in familiar environments, nor draw routes on maps of the familiar environments [13]. However, few studies have formulated theoretical backgrounds in neuronal levels for these symptoms in primates. Recent human behavioral studies suggest that the allocentric spatial knowledge required for navigation in a real environment and drawing routes on a map can be represented as a labeled graph [28],[29]. A labeled graph consists of topological connections (edges) between specific places (nodes), where local metric information, such as distances between nodes (edge weights) and angles between edges (node labels), are incorporated. Here, we propose and discuss that the population activity of primate PCG neurons may represent an environment based on a labeled graph to encode routes in both 3D environments and 2D maps.

## 2. Monkey virtual spatial navigation paradigm

**Figure 1.**
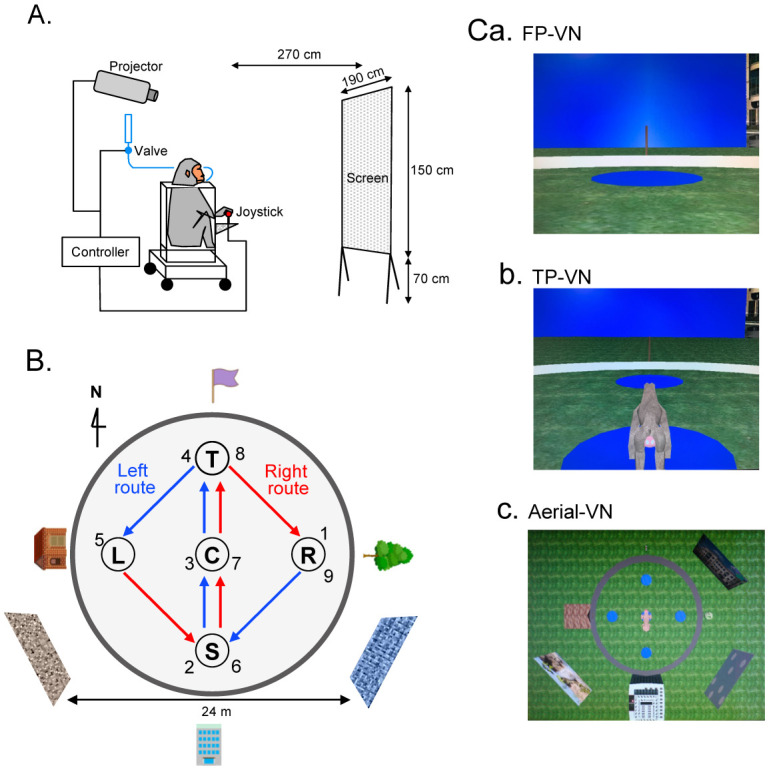
Virtual navigation tasks in monkeys. (A) Setup of the experimental system. (B) Spatial arrangement in and around the mobility area. The monkey was allowed to navigate within the mobility area. In the left route, the monkey visited the reward areas in the order of a series of blue arrows: sequence of reward areas 1→2→3→4→5. In the right route, the monkey visited the reward areas in the order of a series of red arrows: sequence of reward areas 5→6→7→8→9. The common central path segment (location sequence 2-3-4 and 6-7-8) was shared by the left and right routes. (C) Examples of spatial images in the first-person virtual navigation (FP-VN, a), third-person VN (TP-VN, b), and aerial-VN (c) tasks used in this study.

So far, the neuronal basis of representation of the environment based on a labeled graph in the primate PCG is not well understood. This is mainly because few studies investigated primate PCG neuronal activity during 2D and 3D virtual navigation. Thus, to investigate the activity of primate PCG neurons during virtual navigation, we developed a virtual-navigation paradigm in monkeys to test the neuronal basis of labeled graph-based representation of an environment. In this study, a monkey manipulated a joystick in front of a screen on which a virtual environment was projected (Fig. 1A) and navigated along a figure 8-shaped track connecting five reward areas (nodes) in the virtual environments (Fig. 1B). The mobility area where the monkey navigated was surrounded by extra-maze distal cues (Fig. 1B). There were three types of environments for virtual navigation (VN) (Fig. 1C). The monkey was required to navigate in a 3D environment in a first-person view (FP-VN task) (Fig. 1Ca), a third-person view with a monkey avatar (TP-VN task) (Fig. 1Cb), and an aerial view of the environment with a monkey avatar (aerial-VN task) (Fig. 1Cc). Navigation in the aerial view corresponds to route drawing in a 2D map. Here we tested that the population activity of monkey PCG neurons codes the distance traveled along the paths (edge weights) connecting the reward areas (nodes), movement direction (node labels), and movement trajectories in the three different navigational conditions.

The study was performed with one adult male monkey (Macaca fuscata, 6.9 kg). The monkey was kept in a home cage and could freely access rations and fruit or vegetables. Environmental enrichment such as toys was also provided. In the home cage, the animal was deprived of water so that the animal performed the tasks to receive liquid rewards during the experimental session. After each experimental session, additional water and vegetables were provided. The experiment was performed according to the Japan Neuroscience Society Guidelines for the Care and Use of Nonhuman Primates in Neuroscience Research, and the Guidelines for the Care and Use of Laboratory Animals at the University of Toyama. The experimental protocol was approved by the ethical committee for animal experiments at the University of Toyama.

The animal sat in a monkey chair with a stereotaxic frame during the recording session. The monkey's head was then painlessly fixed with the stereotaxic frame by fixing a U-shaped frame that was previously implanted into the animal's skull [30]–[33]. During neuronal recording, the left eye positions of the animal were tracked by an eye-tracking system using a CCD camera with a 150 Hz time resolution [34]. The chair was located 2.7 m away from a screen, on which 3D polarized images were projected from a projector (Fig. 1A). The animal looked at the screen through polarized lenses to perform the VN tasks.

In the FP-VN task, a 3D open space (diameter, 180 m), which was constructed using 3D software (EON Studio ver. 2.5.2, EON Reality, USA), was used [31]–[33] (Fig. 1B). There was a small circular area in the center of the open space (the mobility area: diameter, 24 m) surrounded by a wall. The monkey could freely navigate within the mobility area by manipulating the joystick. Within the mobility area, there were five reward areas (diameter, 2.8 m: S, C, T, L, and R in Fig. 1B). Several extra-maze cues were placed outside the mobility area as the landmark cues. Example images projected to the screen in the FP-VN task are shown in Fig. 1Ca. In this task, to receive liquid rewards, the monkey was required to sequentially visit the reward areas in the fixed sequence; 1) R⇒S⇒C⇒T⇒L (location sequence from 1 to 5; left route), and 2) L⇒S⇒C⇒T⇒R (location sequence from 5 to 9; right route) [33] (Fig. 1B). A liquid reward was provided to the monkey upon entering each reward area in the correct order. Thus, the monkey acquired eight rewards in each trial. It is noted that the same path segment (common central path segment: S⇒C⇒T) was included in both left and right routes. The movements in the common central path segment in the left and right route required the monkey to turn left and right at the T-reward area, respectively. Each PCG neuron was tested with at least ten trials.

In the TP-VN task, the same spatial images were projected to the screen as the FP-VN task, except that the monkey avatar appeared (Fig. 1Cb). The avatar moved depending on the joystick movements, and a reward was obtained when the avatar sequentially entered the reward areas. In the aerial-VN task (avatar translocation task), the virtual space in an aerial view at an angle of 90° (i.e., top view of the movable area) was projected to the screen (Fig. 1Cc). In this task, the monkey moved the avatar in the fixed aerial view by manipulating the joystick so that the avatar sequentially visited the reward areas in the space. The intra-maze cues and routes were identical in all three VN tasks.

Regarding the VN training, the animal was initially trained in the aerial-VN task to manipulate the monkey avatar on the screen. Once the monkey learned the aerial-VN task, the monkey was trained in the TP-VN task with the avatar. After the monkey learned the TP-VN task, the distance from the avatar and camera angle were gradually reduced so that the virtual environment became similar to the FP-VN task. Finally, the monkey learned to perform the FP-VN task from a real-world-like perspective.

## 3. PCG neuronal correlates to spatial information

In this section, we will show that most monkey PCG neurons are responsive to various spatial parameters during VN (i.e., place, routes, head direction, and direction of movements along the path segments). After the monkey learned the three VN tasks, which took approximately one year, the U-shaped acrylic frame, which was fixed to the stereotaxic frame on the monkey chair during recording, was implanted on the skull under aseptic conditions [31]–[33]. The monkey was anesthetized with a combination of ketamine hydrochloride (5.0 mg/kg, i.m.) and medetomidine hydrochloride (0.5 mg/kg, i.m.). The U-shaped frame was fixed with acrylic dental cement to titanium bolts screwed into the skull. The training was resumed two weeks after the surgery while the monkey head was fixed to the stereotaxic frame. Before recording, a tungsten marker electrode calibrated from a reference pin on the head was implanted into the brain under anesthesia based on the stereotaxic atlas [35]. Then, a 3D MRI of the brain was taken to check the stereotaxic coordinates of the recording sites [33]. Finally, a hole for recording was opened in the skull above the PCG under anesthesia. The hole was covered with a Teflon sheet and sealed with epoxy glue until recording.

In the recording session, while the monkey's head was painlessly fixed in the monkey chair, a glass-coated tungsten electrode (Z = 0.5–1.0 MΩ at 1000 Hz) calibrated from the reference pin was stereotaxically inserted into the PCG. After locating neuronal activity, each neuron was tested with the three VN tasks, in which block trials of the three VN tasks were performed in random order. The neuronal activity, triggers for the reward deliveries, X and Y-coordinates of the monkey in the mobility area, joystick movements, and eye positions were digitally stored using a Multichannel Acquisition Processor system (Plexon Inc., Dallas, TX) [33]. The stored data were analyzed by NeuroExplorer software (Nex Technologies, Littleton, MA, USA). The waveforms of neuronal spikes were projected to a feature space, in which each dimension represented a different feature of the waveforms, and manually sorted into single units using Offline Sorter (Plexon Inc., Dallas, TX). Supplementary Figure 1 shows the locations of the 79 PCG neurons (left PCG, n = 43; right PCG, n = 36), derived from 69 penetrations into both hemispheres.

To analyze place responses, the mobility area was divided into 30 × 30 grid pixels [33]. The mean firing rate for each pixel was estimated using data from all visits to that pixel. Then, the firing-rate map was smoothed using a Gaussian function (SD = 1 pixel). Data from the pixel(s), which the monkey did not visit for at least 300 ms in each task, were excluded from the place response analysis. Place-related neurons were defined based on spatial information content (SIC) [36]. SIC was defined as follows:

$$\text{SIC}=\overset{K}{\underset{i=1}{\sum }}Pi\frac{\lambda i}{\lambda }log_{2}\frac{\lambda i}{\lambda }$$ (1)

where *K* is the number of pixels, *Pi* is the occupancy ratio of the *i*th pixel, *λi* is the mean firing rate in the *i*th pixel, and *λ* is the mean firing rate across the pixels. Then, SIC derived from real data of a given neuron and SICs derived from 1000 shuffled surrogates were computed. In the shuffled data, the temporal timing of spikes (inter-spike intervals) was shuffled. Place-related neurons were defined if neurons showed the SIC above the level of chance (p < 0.05). Place fields were also estimated according to the previous study [33] if a given neuron showed place-related responses (see above). To estimate place fields, pixels with a mean firing rate greater than 1.5 times the average firing rates during the task duration were identified. All pixels, which met the criterion (more than 1.5 times the average firing rate) and shared any edge, were combined. Place field(s) was defined when the combined pixels contained at least nine pixels that were visited at least three times during the task.

**Figure 2.**
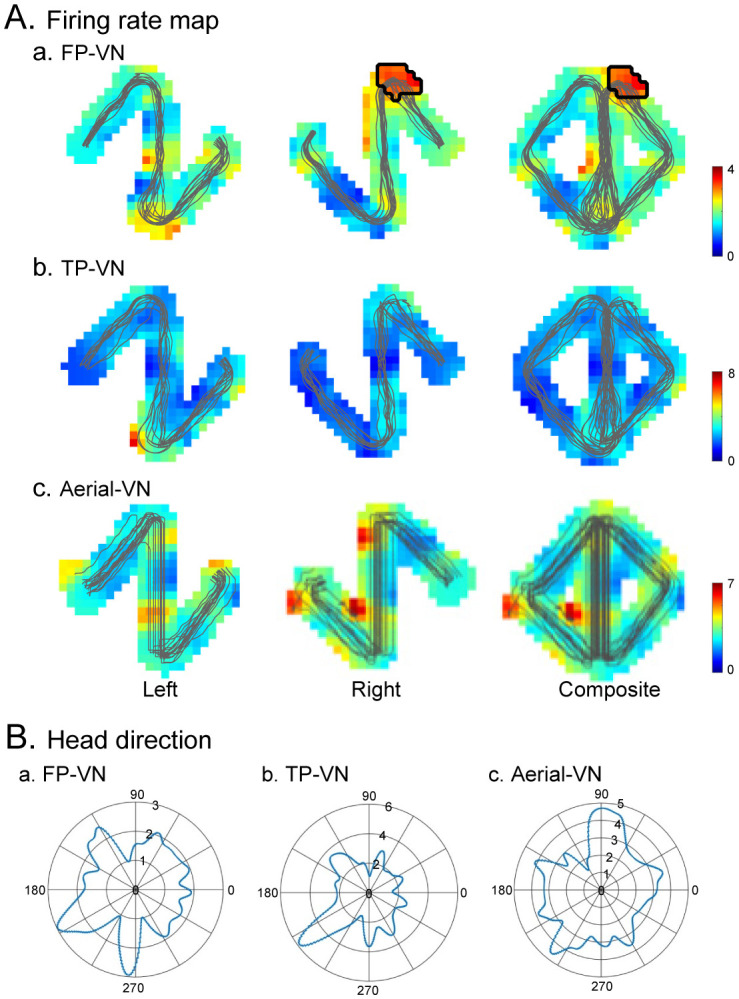
An example of a PCG neuron with place-related (A) and heading direction-differential (B) responses. (A) Firing-rate maps in the FP-VN (a), TP-VN (b), and aerial-VN (c) tasks. Left, left route; Right, right route; Composite, composite routes. In the FP-VN task, this neuron showed place-related responses in the right (b) and composite (c) routes. Thin lines on the firing-rate maps indicate the monkey's movement trajectories. Areas marked by thick lines indicate the place fields. Calibration bars on the right indicate the mean firing rates (spikes/s) in the firing-rate maps. (B) Polar plots of neuronal activity in terms of the head direction (1° of angular bin width) in the FP-VN (a), TP-VN (b), and aerial-VN (c) tasks. Each number on each circle indicates firing activity (spikes/s). This neuron showed differential sensitivity to moving directions in the TP-VN task (Rayleigh test, p < 0.0001).

Of the 79 neurons recorded, 62 (78.5%) displayed place-related responses in at least one of the three tasks (place-related neurons). An example of a PCG-place-related neuron is shown in Fig. 2. Place-related responses were analyzed separately using data from each route and combined routes (right and left routes). In the FP-VN task (Fig. 2Aa), activity increases are observed after passing the T-reward area in the right route but not in the left route. In the composite route, this neuron also showed place-related responses, and a place field was identified in the same area around the T-reward area. However, this neuron showed no place-related activity in the TP-VN and aerial-VN tasks (Fig. 2Ab, c). Thus, this neuron showed place-related responses only in the FP-VN task. In detail, 31 (39.2%, 31/79), 30 (38.0%, 30/79), and 34 (43.0%, 34/79) neurons displayed place-related responses in the FP-VN, TP-VN, and aerial-VN tasks, respectively. Previous studies in rodents have also reported place cells or place-related activity in the RSC [20],[26],[37],[38]. However, few neurons showed significant spatial tuning in the monkey RSC [39], in which monkeys on a stage were passively translocated. Because the RSC and PCC are involved in spatial processing in a large-scale space [40],[41], a small translation on a stage might not affect neuronal activity in the PCG.

**Figure 3.**
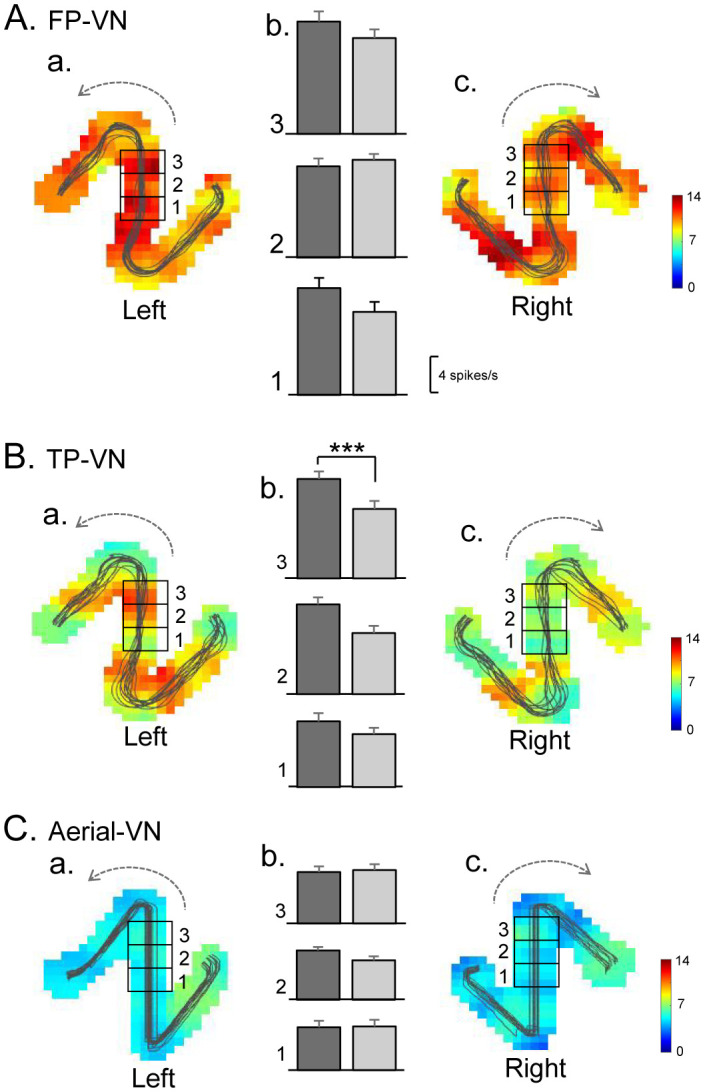
An example of neural activity in the common central path segment with route modulation (a route-modulated zone-related neuron). (A-C) Firing-rate maps in the left (left panel, a) and right (right panel, c) routes in the FP-VN (A), TP-VN (B), and aerial-VN (C) tasks. Thin lines on the firing-rate maps indicate the trajectories of the monkey. The middle panels (b) indicate the mean firing rates in zones 1–3 in the left (dark gray columns) and right (light gray columns) routes. ***, significant main effect of route (p < 0.001).

Second, in previous studies, some neurons differentially respond to places while animals navigate in the same place but on different routes in the rodent anterior thalamic nuclei [42], rodent hippocampus [43]–[46], monkey medial parietal cortex [47], monkey hippocampus [33], and rodent RSC [22],[26],[48],[49]. In the present study, route modulation of place-related activity in the common central path segment was also analyzed according to a previous study [33]. The common central path segment between the C and T reward areas was divided into three zones (zones 1–3). A two-way ANOVA was performed in each VN task to analyze the effect of route and zone on neuronal activity. Route modulation was considered significant (route-modulated zone-related neurons) if PCG neurons showed a significant main effect of the route (p < 0.05) and/or a significant route-zone interaction (p < 0.05). An example of this type of neuron is shown in Fig. 3. In the FP-VN task (Fig. 3A), there was no significant main effect of the route in the common central path segment [F(1,57) = 2.353, p = 0.1305] nor a significant interaction between the route and zone [F(2,57) = 1.546, p = 0.2218]. However, in the TP-VN task, there was a significant main effect of route [F(1,63)=15.25, p = 0.0002] but no significant interaction between route and zone [F(2,63) = 0.8468, p = 0.4336] (Fig. 3B). In the aerial-VN task, there was no significant main effect of route [F(1,69) = 0.2637, p = 0.6092] nor a significant interaction between route and zone [F(2,69) = 0.6952, p = 0.5024] (Fig. 3C). These findings indicate that this PCG neuron showed route-modulated zone-related responses only in the TP-VN task. Of the 79 PCG neurons tested, 42 displayed route-modulated zone-related responses in at least one of the VN tasks. Thus, the monkey PCG neurons displayed route-modulated responses in the common central path segment, consistent with the previous rodent RSC studies. Interestingly, the silencing of these neurons in the mouse RSC disturbed task performance in a T-maze [49], suggesting that these neurons are involved in motor planning for correct navigation in a given route. In humans, the RSC and PCC were also reported to be active during route planning [8],[12].

Third, previous studies have reported various types of head direction cells in the rodent RSC [18]–[21],[50] and movement-direction-sensitive cells in the RSC [22]. To analyze responsiveness to specific head directions of the monkey (FP-VN task) and avatar (TP-VN and aerial-VN tasks), polar plots (1° angular bin width) of neuronal activity in terms of specific head directions were constructed. In the aerial-VN task, the head direction of the avatar in the allocentric 3D virtual space, but not on the screen, was used. The resulting tuning curves were smoothed using a Gaussian kernel (SD = 6°). A significant deviation in each polar plot was tested using the Rayleigh test, and head direction-differential neurons were defined if the neurons showed significant differences (p < 0.05). Examples of head direction tuning curves in one neuron are shown in Fig. 2B (the same neuron shown in Fig. 2A). This neuron showed differential sensitivity to head directions in the TP-VN task (Rayleigh test, p < 0.0001) but not in the FP-VN (Rayleigh test, p = 0.281) and aerial-VN (Rayleigh test, p = 0.473) tasks. Of the 79 neurons tested, the activity of 71 differentially increased when the monkey or avatar faced certain directions (Rayleigh test, p < 0.05). However, directional tuning of these monkey PCG neurons was broad and not unimodal, indicating that these PCG head direction-differential neurons were not classic head direction cells. The RSC is implicated in the integration of self-motion signals such as locomotion and vestibular information from the subcortical head direction system (e.g., thalamus) and cortical visual signals such as landmarks [51]. In contrast, vestibular information is particularly important for generating head direction signals in the thalamic head direction system [52]. These findings suggest that the broad tuning of the monkey PCG neurons to the head direction might be attributed to a lack of vestibular information during VN.

**Figure 4.**
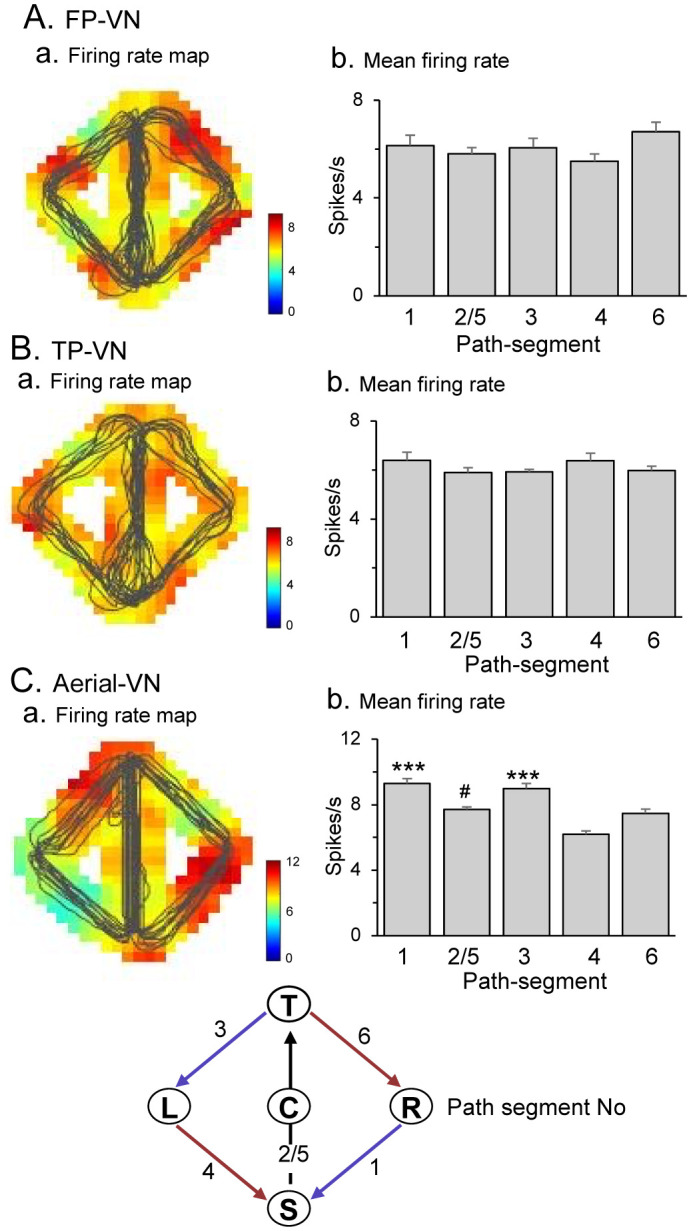
An example of a path segment-differential neuron. (A-C) Firing-rate maps (a) and mean firing rates in the five segments (b) in the FP-VN (A), TP-VN (B), and aerial-VN (C) tasks. ***, significant difference from the path segments 2/5, 4 and 6 (Tukey test, p < 0.001); #, significant difference from the path segment 4 (Tukey test, p < 0.01).

We also analyzed the differential sensitivity of PCG neurons to directional movements along the five path segments separated by the reward deliveries (common central path segment between the S - T-reward areas and path segments between the T - R, R - S, T - L, and L - S reward areas). The mean firing rates during navigation between these reward deliveries were compared among the five linear path segments using a one-way ANOVA. Path segment-differential neurons were defined if the neurons showed a significant difference among the five path segments (p < 0.05). An example of a path segment-differential neuron is shown in Fig. 4. In the FP-VN and TP-VN tasks (Fig. 4A, B), there were no significant differences in the activity of this neuron among the five path segments [Fig. 4A: F(4,70) = 1.463, p = 0.2228; Fig. 4B: F(4,52) = 1.096, p = 0.3685]. However, in the aerial-VN task (Fig. 4C), there were significant differences in neuronal activity among the five path segments [F(4,64) = 24.36, p < 0.0001]. The activity of this neuron was significantly greater in path segments 1 and 3 than in other path segments (Tukey test, p < 0.001). Of the 79 PCG neurons tested, 57 neurons showed path segment-differential responses in at least one of the three VN tasks. These data suggest that the primate PCG neurons code movements along the linear path segment, consistent with the rodent RSC data [22].

Thus, the PCG neurons were active during VN. This suggests that the spatial activity of the PCG during the task is attributed to visual inputs, such as optic flow, as well as specific spatial views. Consistent with the present results, the RSC receives visual afferents from the cortical (areas 17 and 18b) and thalamic visual areas [53],[54], whereas the PCC receives visual information from the parietal cortex [55],[56]. In rodents, spatial cue lights activate RSC neurons [48], whereas the spatial activity of RSC neurons is suppressed in the dark [22]. Furthermore, RSC lesions make rats less sensitive to distal visual cues to control behavioral performance [57]. In humans, activity in the RSC increases when the head direction is estimated from the optic flow [58]. Furthermore, it has been reported that visual inputs are sufficient to form a memory of large-scale spaces, such as a cognitive map in humans [59]. In the present study, successive navigation in the virtual spaces suggests that the monkey may form a cognitive map in the brain based on virtual navigation-sensitive neurons (see below).

## 4. PCG neural correlates to rewards

**Figure 5.**
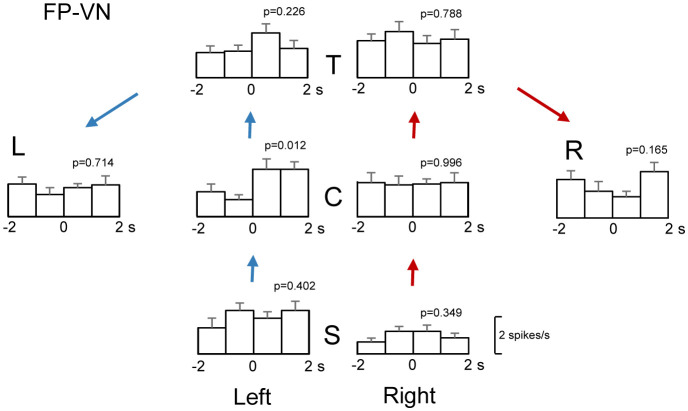
An example of reward-related responses with route modulation (a route-modulated reward-related neuron). This neuron showed a significant interaction between the route and reward area (p = 0.0021). Each histogram indicates the mean firing rates before and after reward delivery. Zero on the abscissa indicates the time point of reward delivery. Each p-value indicates the results of a one-way ANOVA in each reward area.

Responses to rewards in this paradigm are important to encode nodes in a labeled graph-based environment. In the hippocampus, two types of neurons are proposed to play an important part in the navigation to a goal as well as episodic memory: place cells code the subject's locations while reward-related neurons code the locations of goals (goal-directed cells) [60]–[62]. These findings suggest that receiving rewards at fixed locations (i.e., nodes in this paradigm) contributes to the updating of one's position and consequently contributes to navigation. To investigate PCG neuronal responses to reward delivery in each reward area, a peri-event histogram in 1-s bins during 4 s around reward delivery (each 2 s before and after the reward delivery) was constructed in each reward area. These data were analyzed with a one-way ANOVA: reward-related neurons were defined when significant differences among the four 1-s bins were observed at least at one of the reward areas in either route [33]. An example of a reward-related PCG neuron is shown in Fig. 5. This neuron displayed reward-related responses at the C-reward area in the left route: there was a significant difference among the four 1-s bins in this neuron [F(3,52) = 4.012, p = 0.0121]. The 74 of the 79 PCG neurons displayed reward-related responses in at least one of the reward areas in at least one of the three VN tasks. Consistent with the current results indicating high sensitivity of the PCG neurons to reward deliveries, the reward is one of the crucial variables affecting neuronal activity in the rodent RSC [48],[63],[64] as well as in the monkey PCC [65],[66]. Furthermore, in 7 of the 74 reward-related neurons, reward-related responses were observed at specific reward areas only in one specific VN task, while reward-related responses were observed in multiple VN tasks in the remaining 67 neurons. In these 67 neurons, locations of the reward areas with the reward-related responses were different across the VN tasks. Thus, reward-related responses were observed only in some specific reward areas, locations of which were different across the VN tasks, suggesting that these neurons may be involved in the navigation to specific reward areas (i.e., graph nodes) in the specific VN environments. In the rodent hippocampus, some neurons encoded locations of specific reward areas differently across different environments [67]. The present results in the PCG were consistent with the data in the rodent hippocampus [67]. These findings further suggest that these reward-related neurons might contribute to the encoding of contexts across the three VN tasks.

A previous study in the monkey hippocampus reported that responses to rewards were route-dependent [33]. In this study, route modulation of reward-related responses in the common central path segment was also examined: a three-way ANOVA was performed to analyze the effects of the route (left vs. right route), reward area (T vs. C vs. S), and period (four 1-s periods around the reward delivery) on the mean firing rates. Route modulation of reward responses (route-modulated reward-related neurons) was defined if there were a significant main effect of the route and/or significant interaction between the reward area and route, a significant interaction between period and route, or significant interaction among reward area, route, and period (p < 0.05). An example of a PCG neuron with a significant route modulation of reward-related responses is shown in Fig. 5. This neuron displayed a significant interaction between the route and reward area [F(2,312) = 6.29, p = 0.0021]. Of 79 PCG neurons, 57 displayed route-modulated reward-related responses in the common central path segment. These route-modulated reward-related neurons may be also involved in the coding and planning of routes.

## 5. Decoding of labeled graph-based spatial information

Since our results described in the sections above indicate that monkey PCG neurons are sensitive to various spatial parameters during VN, we examined whether spatial parameters could be decoded from the ensemble activity of PCG neurons. A previous neurophysiological study reported that changing a linear running track to folded zigzags or insertion of a protruded loop into the running track, which did not alter the topology of starting and goal locations, did not affect the spatial responses of hippocampal place cells if the spatial responses in the altered running tracks were fitted to its original linear track, suggesting that those hippocampal place cells code topology of places [68]. In the same way, actual spatial parameters and PCG neuronal activity were rescaled to fit navigation along the left and right routes consisting of the linear path segments as assumed in the labeled graph of the virtual space. Then, mean firing-rate maps along the left and right routes were constructed in each task (192 timestamps across the left and right routes).

**Figure 6.**
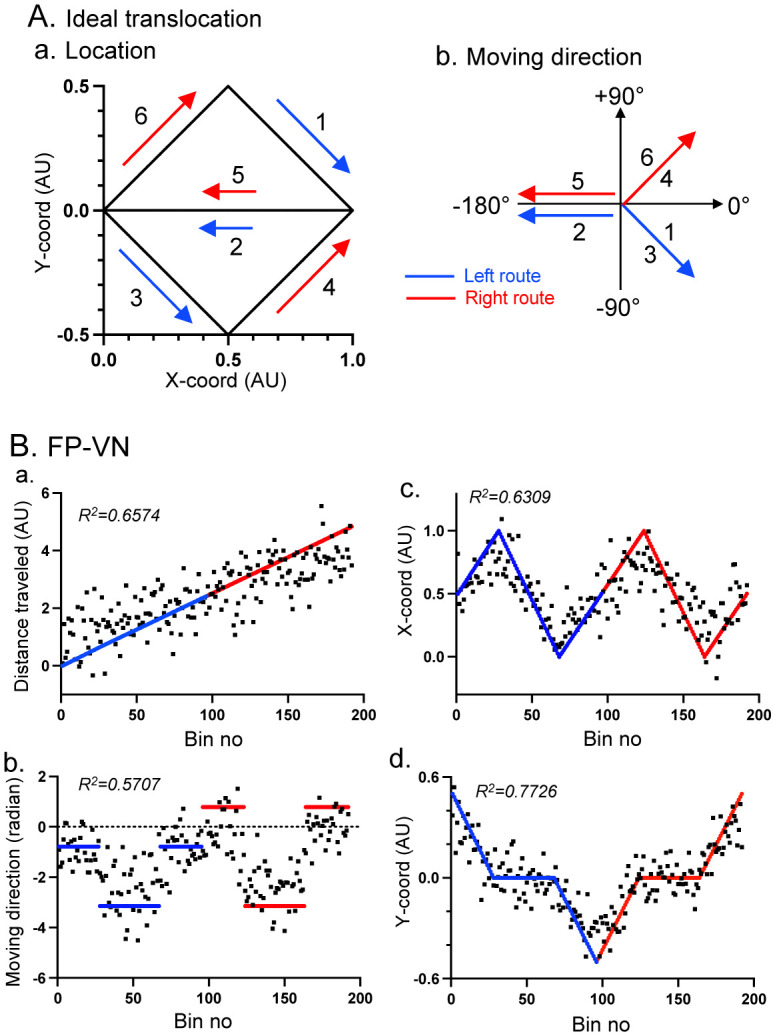
Decoding of the spatial parameters from the data in the FP-VN task. (A) Ideal location (Aa) and moving direction (Ab) of the monkey and avatar during the tasks based on the labeled theory. (B) Predicted (black dots) and actual ideal (blue and red lines) spatial parameters [distance traveled (a), moving direction (b), X-coordinates (c), and Y-coordinates (d)] in the FP-VN task. AU, arbitrary unit.

A previous rodent study reported that each spatial parameter (position, speed, and context) could be individually decoded from the ensemble activity of RSC neurons using multiple linear regression analysis [38]. To decode each spatial parameter along the left and right routes [X- and Y-coordinates of one's own or avatar's location, distance traveled from the starting point in the left route (R reward area) to the endpoint in the right route (R reward area), and moving direction in radian], relationships between ensemble activity of the 79 PCG neurons and each parameter were analyzed in each VN task using multiple linear regression analysis (Prism 9, GraphPad Software, LLC.) with the following equation:

$$\text{Predicted value in each parameter at }i\text{-th time bin}=\beta_{0}+\sum^{\text{​}}\beta_{j}R_{ij}\text{ for }j=\text{neuron number}\left(\text{from 1 to 79}\right)$$ (2)

where *β_0_* is offset, *R_ij_* is firing rates of neuron *j* at *i-*th time bin, and *β_j_* are weights for neuron *j* that minimize the differences between the actual and predicted values across all time bins. Figure 6A shows the ideal location (Aa) and moving direction (Ab) of the monkey during the tasks as assumed in the labeled graph of the virtual spaces, which actual spatial parameters [distance traveled, location (X- and Y-coordinates), and moving direction] were rescaled to fit. Figure 6B shows the predicted (black dots) and actual ideal (blue and red lines) spatial parameters [distance traveled (Ba), moving direction (Bb), X-coordinates (Bc), and Y-coordinates (Bd)] in the multiple linear regression analyses in the FP-VN task. The results derived from the linear regression model indicated accurate decoding of the spatial parameters of the ideal translocation. To analyze whether the linear regression model could extract meaningful information, we created 1000 shuffled surrogates in which timestamps of the data were shuffled within each trial consisting of 192 time bins using a custom script written in MATLAB (MathWorks, Inc., Natick, MA). The same analyses of the shuffled data indicated that the degrees of the data fitting (R^2^) were significantly larger in the rescaled real data than those in the shuffled data in the FP-VN task (p < 0.001).

**Figure 7.**
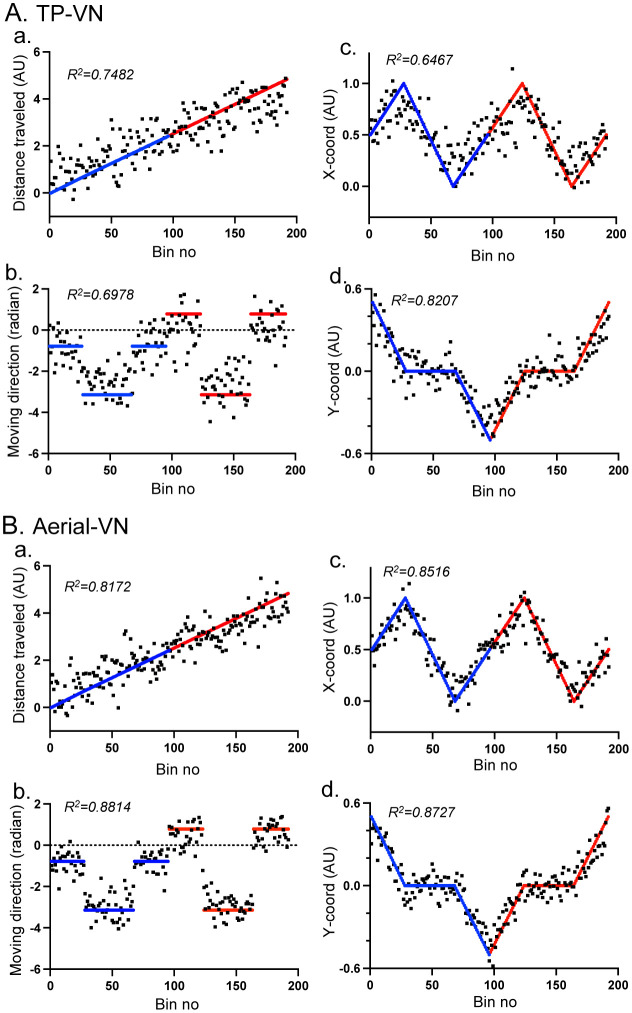
Decoding of the spatial parameters [distance traveled (a), moving direction (b), X-coordinates (c), and Y-coordinates (d)] from the data in the TP-VN (A) and aerial-VN (B) tasks. AU, arbitrary unit. Other descriptions as for Fig. 6.

Figure 7A shows the same decoding analyses in the TP-VN task. The comparable data were observed with higher degrees of the data fitting (R^2^). The comparison of these data with those in the shuffled data indicated that the degrees of the data fitting (R^2^) were significantly larger in the rescaled real data than those in the shuffled data in the TP-VN task (p < 0.001). Figure 7B shows the same decoding analyses in the aerial-VN task. The same comparable data were observed with higher degrees of data fitting (R^2^) than those in the TP-VN task. The degree of the data fitting (R^2^) was significantly larger in the rescaled real data than in the shuffled data in the aerial-VN task (p < 0.001). It is noted that, for decoding of moving direction in the aerial-VN task, the actual ideal moving direction in the FP-VN and TP-VN tasks was used. This indicates that moving direction in the FP-VN and TP-VN tasks was accurately decoded from the data in the aerial-VN task.

Thus, the preliminary results derived from one monkey indicated that the ensemble activity of the PCG neurons represents the spatial parameters during VN in the labeled graph-based environments. Patients and rodents with PCG (RSC and/or PCC) lesions cannot navigate in familiar environments, and those patients cannot draw routes on 2D maps of the familiar environments (see Introduction), suggesting that the representation of allocentric space to find the right route to a destination is impaired in subjects with PCG lesions. One way to represent the locations and routes connecting these locations in both 3D environments and 2D maps is a topological graph or cognitive graph [68],[69], which has been suggested in human and rodent studies [28],[29],[68],[70]–[72]. We hypothesized that the PCG encodes labeled graphs of the locations and routes in the environment. It appears that the population activity of the PCG neurons represents the distance traveled, locations, movement direction, and routes in each task. Consistently, the ensemble activity of RSC neurons encodes multiple parameters, including the locations and speeds of animals as well as task contexts in mice [38]. Furthermore, movement directions in the 3D environment in the FP-VN and TP-VN tasks can be estimated from the ensemble neuronal activity during the aerial-VN task that corresponds to the 2D map. These findings imply the existence of a labeled graph-based representation of the environment in the PCG.

## 6. Conclusions and discussion

The analyses of the individual PCG neurons indicated that the PCG neurons responded to specific spatial parameters including place, head direction, and path segment. It has been suggested that such spatial processing is subserved by sparse coding or continuous attractor computation based on an interaction between pyramidal neurons and interneurons through lateral and feedforward inhibition as well as feedback inhibition in rodents [17],[73],[74]. Consistently, there are at least two types of neurons (pyramidal neurons and interneurons) in the rodent RSC, and local neural circuits in the rodent RSC are dominated by feedforward inhibition [75]. Previous studies classified neuronal activities into putative pyramidal neurons and interneurons based on the spike widths (peak-to-trough distance) and/or firing rates, using k-means clustering [76],[77]. In the present study using the same neurophysiological classification methods based on spike widths, the 79 PCG neurons were classified into 70 putative pyramidal neurons and 9 putative interneurons (Supplementary Figure 2). These findings suggest that spatial processing in the primate PCG might be subserved by similar neural circuits to those in the rodent RSC.

The decoding results suggest that labeled graph-based environments are represented in the PCG. Human fMRI studies suggest that the PCG encodes the locations of scene images and distances between scene images in the real environment and the facing direction of subjects in the real environment in response to scene images [78],[79], and is active during mental imagery of self-rotation [80]. Furthermore, the RSC is active during path integration tasks, and path integration accuracy is correlated with the gray matter volumes of the RSC [81],[82]. These human data are consistent with the current results that were analyzed according to the labeled graph hypothesis. The representation of the allocentric environment by a topological graph supports the transformation between egocentric and allocentric information, which is postulated in the PCG [2],[80],[83]–[85]. We suggest that the ensemble activity of PCG neurons supports these spatial functions, such as the schematic representation of the environment [86].

A computational study suggested that the hippocampal CA3 area represents allocentric space based on a topological graph, in which cells coding for specific places (nodes) are connected with other place cells coding for different places. The connection weights between the nodes (edges) are proportional to the synaptic resistance (distance between places) [69]. Thus, allocentric information can be encoded in the networks of nodes connected to each other in the hippocampus. On the other hand, the development of spatial coding is relatively slow in the RSC compared with the hippocampus in rodents, and the emergence of spatial coding in the RSC requires an intact hippocampus [87],[88]. In humans, comparative findings have been reported: the PCG, including the RSC and PCC, is active during the processing of spatial information in a familiar environment, whereas the hippocampus is active during learning of a new environment [7],[86],[89]. Furthermore, functional connectivity between the PCC and the parahippocampal cortex which has intimate anatomical connections to the hippocampus is positively correlated with performance of memory encoding and retrieval [90],[91]. These findings imply that hippocampal allocentric representation by a cognitive graph might be gradually transferred to the PCG.
