# Supplementary material for: Neural basis of topographical disorientation in the primate posterior cingulate gyrus based on a labeled graph
Source: AIMS Neurosci. 2022 Sep 9;9(3):373–94. doi: 10.3934/Neuroscience.2022021 (PMC9581735; doi:10.3934/Neuroscience.2022021)
Supplement: Supplementary file 1 [file neurosci-09-03-021-s001.pdf]

AIMS Neuroscience, 9(3): 373–394.
DOI: 10.3934/Neuroscience.2022021
Received: 23 June 2022
Revised: 29 August 2022
Accepted: 06 September 2022
Published: 09 September 2022

http://www.aimspress.com/journal/neuroscience

Theory article

Neural basis of topographical disorientation in the primate posterior cingulate gyrus based on a labeled graph

Yang Yu [1], Tsuyoshi Setogawa[1,2], Jumpei Matsumoto[1,2], Hiroshi Nishimaru[1,2] and Hisao Nishijo [1,2,*]

[1] System Emotional Science, Faculty of Medicine, University of Toyama, Toyama, Japan
[2] Research Center for Idling Brain Science (RCIBS), University of Toyama, Toyama, Japan

* **Correspondence:** Email: nishijo@med.u-toyama.ac.jp; Tel: +81-764347215;
Fax: +81-764345012.

Supplementary materials

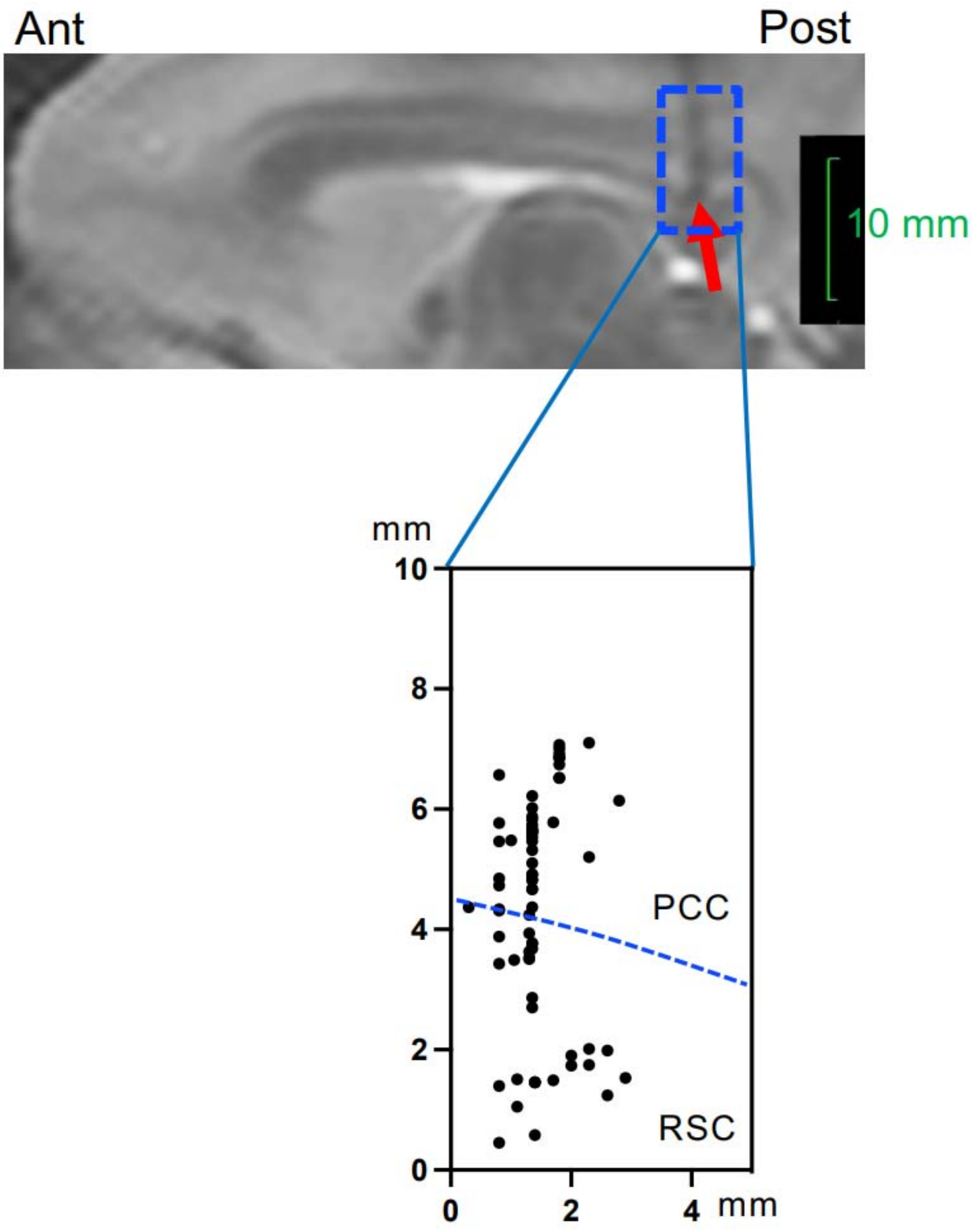

Supplementary Figure 1. Recording sites in the PCG. Recording sites (dots) of PCG neurons are plotted in the sagittal plane of the brain. A red arrow indicates a tip of a marker electrode. Ant, anterior; Post, posterior.

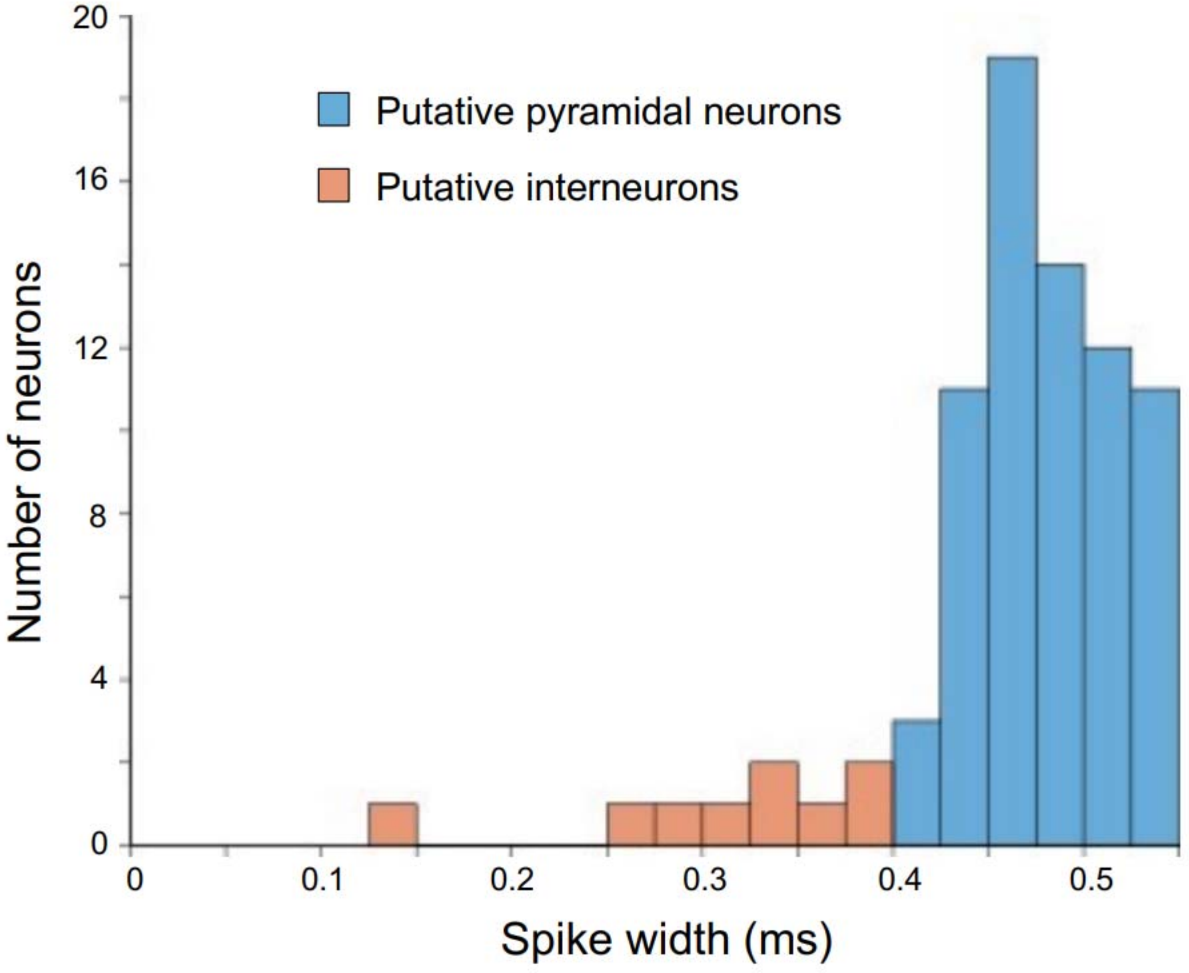

Supplementary Figure 2. Classification of putative pyramidal neurons (blue columns) and interneurons (brown columns) using k-means clustering. Spike widths were used for k-means clustering with the number of clusters specified as 2.

AIMS Press

© 2022 the Author(s), licensee AIMS Press. This is an open access article distributed under the terms of the Creative Commons Attribution License (http://creativecommons.org/licenses/by/4.0)
